# Increased Levels of Omega-3 Fatty Acids and DHA Are Linked to Pain Reduction in Rheumatoid Arthritis Patients Treated with Janus Kinase Inhibitors

**DOI:** 10.3390/nu13093050

**Published:** 2021-08-30

**Authors:** Ching-Kun Chang, Po-Ku Chen, Chia-Ching Chen, Shih-Hsin Chang, Chu-Huang Chen, Der-Yuan Chen

**Affiliations:** 1 Rheumatology and Immunology Center, China Medical University Hospital, Taichung 404, Taiwan; kun80445@gmail.com (C.-K.C.); pago99999@gmail.com (P.-K.C.); sherry61976@hotmail.com (S.-H.C.); 2 Translational Medicine Laboratory, China Medical University Hospital, Taichung 404, Taiwan; 3 College of Medicine, China Medical University, Taichung 404, Taiwan; 4 School of Medicine, Chang Gung University, Tao-Yuan 333, Taiwan; daminglife@gmail.com; 5 Ph.D. Program in Translational Medicine and Rong Hsing Research Center for Translational Medicine, National Chung Hsing University, Taichung 402, Taiwan; 6 Department of Life Innovation, Institute for Biomedical Sciences, Shinshu University, Matsumoto 390-8621, Japan; cchen@texasheart.org; 7 Vascular and Medicinal Research, Texas Heart Institute, Houston, TX 77030, USA; 8 New York Heart Research Foundation, Mineola, New York, NY 11501, USA

**Keywords:** omega-3 fatty acids, docosahexaenoic acid (DHA), analgesic effect, rheumatoid arthritis, Janus kinase inhibitors

## Abstract

Although Janus kinase inhibitors (JAKi) could reduce patient-reported pain in rheumatoid arthritis (RA), their mechanism remains unclear. Therefore, we examined lipid metabolites change in JAKi-treated patients and evaluate their association with pain reduction. We used ^1^H-NMR-based lipid/metabolomics to determine serum levels of lipid metabolites at baseline and week 24 of treatment. Serum levels of significant lipid metabolites were replicated by ELISA in 24 JAKi-treated and 12 tocilizumab-treated patients. Pain was evaluated with patients’ assessment on a 0–100 mm VAS, and disease activity assessed using DAS28. JAKi or tocilizumab therapy significantly reduced disease activity. Acceptable pain (VAS pain ≤20) at week 24 was observed in 66.7% of JAKi-treated patients, and pain decrement was greater than tocilizumab-treated patients (ΔVAS pain 70.0 vs. 52.5, *p* = 0.0595). Levels of omega-3 fatty acids and docosahexaenoic acid (DHA) were increased in JAKi-treated patients (median 0.55 mmol/L versus 0.71 mmol/L, *p* = 0.0005; 0.29 mmol/L versus 0.35 mmol/L, *p* = 0.0004; respectively), which were not observed in tocilizumab-treated patients. ELISA results showed increased DHA levels in JAKi-treated patients with acceptable pain (44.30 µg/mL versus 45.61 µg/mL, *p* = 0.028). A significant association of pain decrement with DHA change, not with DAS28 change, was seen in JAKi-treated patients. The pain reduction effect of JAKi probably links to increased levels of omega-3 fatty acids and DHA.

## 1. Introduction

Rheumatoid arthritis (RA) is characterized by inflammation and hyperplasia of synovia, cartilage degradation, and bone erosions [1,2]. Pain, a dominant component of the patient-reported outcome, can significantly burden RA patients’ quality of life. Therefore, reduction of RA-related pain is one major need of patients [3,4]. Pain associated with RA is multifactorial and complex [5,6,7,8,9,10], and the mechanisms include peripheral joint inflammation, noninflammatory nociceptive stimuli, peripheral/central sensitization, and JAK/STAT pathway [5,6,7,8,9,10]. Although the current biologics could effectively control RA-related inflammation, as reflected in the recent management recommendations [11], nearly one-third of patients reported unacceptable pain after 21 months of combination therapy [12]. Furthermore, Rifbjerg-Madsen et al. demonstrated that more than 50% of the Danish arthritis patients reported persistent pain despite inflammation control [13]. These observations indicate insufficient effects of the current therapeutic strategies, including biologics, on noninflammatory nociceptive pain in RA patients.

Janus kinase inhibitors (JAKi) exert their therapeutic effects by blocking JAK/STAT-mediated signaling implicated in RA pathogenesis. The JAKi tofacitinib (a JAK1/JAK3 inhibitor) and baricitinib (a JAK1/JAK2 inhibitor) are effective in RA treatment [14,15,16]. Given the increasingly acknowledged implication of the JAK/STAT pathway in the modulation of pain and nociceptive response [9,10], JAKi have an additional beneficial effect of pain reduction in RA patients. Several clinical trials have revealed that tofacitinib additionally produced sustained pain reduction in RA [17,18,19]. Similarly, the RA-BEAM Trial demonstrated that baricitinib therapy provided greater pain reduction than adalimumab and placebo groups [20]. Among RA patients with low disease activity, the average improvements in pain scores were significantly greater in baricitinib-treated patients compared with placebo group [21]. With a matching-adjusted indirect comparison, Fautrel et al. revealed greater pain reduction after baricitinib monotherapy compared with tocilizumab (TCZ) or adalimumab monotherapy, while no significant difference in pain decrement between baricitinib and tofacitinib [22]. These findings suggest that JAKi, either tofacitinib or baricitinib, could additionally reduce RA-associated pain, and the underlying mechanism is worth further exploration.

Lipid metabolomics, relatively recent research, could be employed to characterize lipid metabolites and investigate their biological roles in lipid metabolism [23]. ^1^H nuclear magnetic resonance (NMR)-based lipidomics has been used for diagnosis or therapeutic response follow-up [24]. Souto-Carneiro et al. revealed a distinct lipidomic signature in seronegative RA using the ^1^H NMR-based lipidomics [25]. Among the lipid metabolites, omega-3 polyunsaturated fatty acids (PUFAs) possess anti-inflammatory and analgesic properties [26], and docosahexaenoic acid (DHA) is the precursor of potent anti-inflammatory mediators such as resolvins and protectins [27]. These lipid mediators could attenuate inflammatory pain through central and peripheral actions [28]. Many studies, including a systemic review and meta-analysis, revealed that omega-3 PUFAs and DHA might reduce more pain in RA patients, either compared with baseline or placebo [29,30]. Therefore, we speculate a possible association of these lipid metabolites with pain reduction in JAKi-treated patients.

In this prospective pilot study, we used ^1^H NMR-based lipid/metabolomics to investigate the changes in serum levels of omega-3 PUFAs and DHA in RA patients treated with JAKi or TCZ and utilized ELISA to perform a replication study on the significant lipid metabolites. Since a meta-analysis revealed similar lipid profile changes in patients treated with JAKi or TCZ [31], we enrolled TCZ as the control medication. Besides, we examined the correlation between the changes of lipid metabolites levels and the decrement of pain scores in RA patients treated with JAKi or TCZ.

## 2. Materials and Methods

### 2.1. Patients and Study Design

In this prospective study, we randomly enrolled 36 active RA patients who fulfilled the 2010 classification criteria of the American College of Rheumatology/European League Against Rheumatism collaborative initiative [32] and were available for examination of lipid metabolites before and after six months’ JAKi or TCZ therapy. Disease activity was assessed using the 28-joint disease activity score (DAS28) [33], with active status defined as a DAS28 > 3.2. Thirty-six biologic-naïve, active RA patients who had received conventional synthetic disease-modifying antirheumatic drugs (csDMARDs) started JAKi (tofacitinib or baricitinib, *n* = 24) or IL-6R inhibitor (TCZ, *n* = 12) therapy according to the guidelines [34]. Patients assessed the pain on a 0-100 mm visual analogue scale (VAS) at baseline and week 24 of JAKi or TCZ treatment. The Institutional Review Board approved this study (CMUH109-REC3-161), with each participant’s written consent obtained according to the Declaration of Helsinki.

### 2.2. The Major Outcome for Pain

The major outcome for pain is the proportion of patients reporting pain scores equal to or less than 20 (VAS pain ≤ 20 mm) at week 24, so-called “acceptable pain” [12]. Pain scores ≤ 20 mm threshold represent a threshold when human satisfaction with health is not negatively influenced by pain [35]. In contrast, “unacceptable pain” is defined as the VAS pain scores more than 20 mm despite inflammation control.

### 2.3. Blood Sample Preparation and Lipid Profiles Measurement

Overnight-fasted venous blood samples were obtained in the morning and stored at −80 °C until use. Plasma levels of total cholesterol, triglyceride, high-density lipoprotein cholesterol (HDL-c), and low-density lipoprotein cholesterol (LDL-c) were measured using enzymatic methods with a chemistry analyzer AU5800 (Beckman Coulter, Brea, California, USA) according to the manufacturer’s instructions.

### 2.4. Determination of Serum Lipid Metabolites by ^1^H-NMR Lipid/Metabolomics

A serum sample was analyzed using the ^1^H-NMR lipid/metabolomics (Nightingale Health, Helsinki, Finland) [23,24], with 100μl serum and phosphate buffer (prepared with 5.5 mM sodium 3-trimethylsilyl (2,2,3,3-d4) propionate, 0.075 molarity Na_2_HPO_4_·7H_2_O, 5 mL NaN_3_ (4%) adjusted to pH 7.4 with 1 M HCl) mixed in an Eppendorf tube. The complete sample was transferred to a 3 mm NMR tube (Bruker Match system) and measured at 310 K in Bruker Avance III NMR spectrometers operating at 600.13 MHz equipped with a maximum gradient strength of 53 G/cm. Each sample was equilibrated at 310 K for 5 min before data acquisition. Each data set was automatically processed using a line broadening of 1Hz, with the NOESY data aligned to the alanine signal at 1.49 ppm. The whole lipid/metabolites was list in Supplemental Table S1.

### 2.5. Quantification and Replication of the Significant Lipid Metabolites Using ELISA

Among the changes in lipid metabolites analyzed by lipid/metabolomics, serum levels of omega-3 fatty acids and DHA were significantly increased in JAKi-treated patients. We quantified serum DHA levels with the available commercial ELISA kit (MyBioSource, San Diego, CA, USA) following the manufacturer’s instruction. The coefficient of variation (CV%) for the internal standards was less than 10%.

### 2.6. Statistical Analysis

The results were presented as the mean ± standard deviation (SD) or the median (interquartile range, IQR). The nonparametric Mann–Whitney U test was used for between-group comparisons of numerical variables. Wilcoxon signed-rank test was employed to compare serum levels of lipid metabolites during follow-up in patients after a six-month therapy. The correlation coefficient was calculated using the nonparametric Spearman’s rank correlation test. A two-sided *p*-value < 0.05 was considered statistically significant.

## 3. Results

### 3.1. Demographic Data and Clinical Characteristics of RA Patients

**Figure 1:**
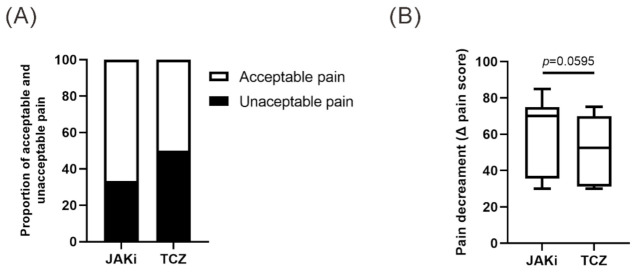
(**A**) Proportion of acceptable pain and unacceptable pain and (**B**) comparison of pain decrement in JAKi-treated and TCZ-treated patients. A Fisher’s exact test was used for between-group comparison of the proportion of acceptable pain. Data in (**B**) are presented as box-plot diagrams, with the box encompassing the 25th percentile (lower bar) to the 75th percentile (upper bar). The horizontal line within the box indicates median value respectively for each group. The *p*-value was determined by the Mann–Whitney U test. JAKi: Janus kinase inhibitors; TCZ: tocilizumab.

As illustrated in Table 1, JAKi or TCZ therapy significantly decreased disease activity (DAS28) assessed at week 24. We observed nonsignificant higher proportion of patients reporting “acceptable pain,” pain scores (VAS ≤ 20 mm), at week 24 in JAKi-treated patients compared with TCZ-treated patients (66.7% versus 50.0%, Figure 1A). The pain decrement was also greater in JAKi-treated patients than in those treated with TCZ (ΔVAS pain 70.0 vs. 52.5, *p* = 0.0595, Figure 1B). As revealed in Table 1, there were no significant differences in demographic data, clinical characteristics, the proportion of positivity for rheumatoid factor or anticitrullinated peptide antibody, disease activity at week 24, the change of lipid profiles, the proportion of concomitant medications or comorbidities between JAKi-treated and TCZ-treated patients.

### 3.2. Change in Serum Levels of Omega-3 PUFAs and DHA Determined the ^1^H NMR-Based Lipid/Metabolomics in Patients Treated with 6-Month JAKi or TCZ

**Figure 2:**
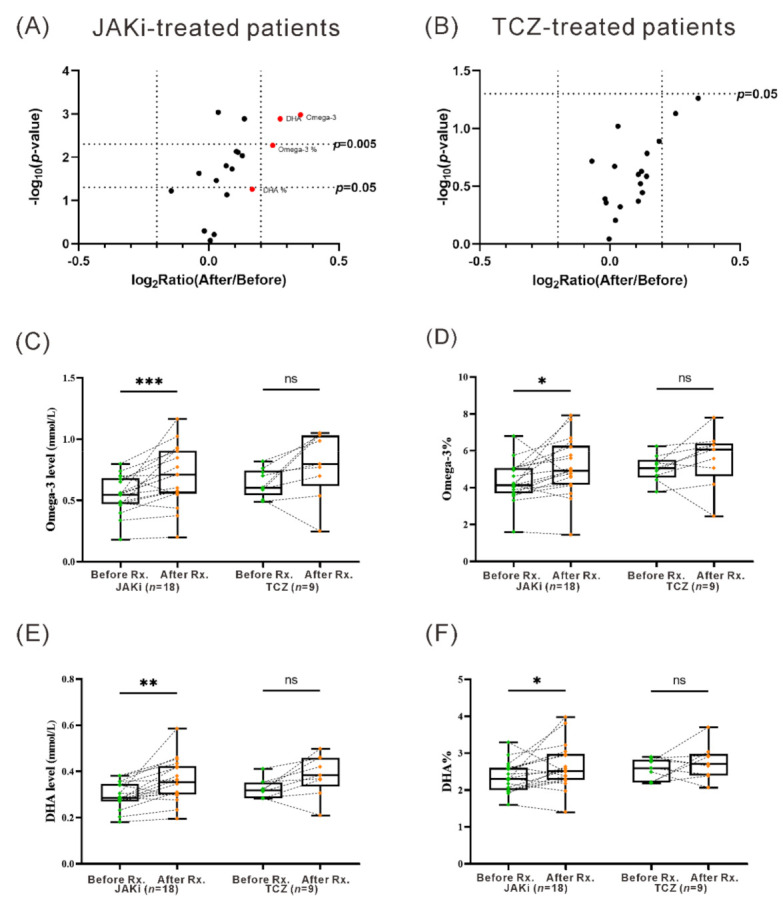
The change in serum levels of lipid metabolites determined by the ^1^H NMR-based lipid/metabolomics in patients treated for six months with JAKi or TCZ. The volcano plot of fatty acids levels changes in (**A**) JAKi-treated or (**B**) TCZ-treated patients. The comparison of serum levels of (**C**) omega-3 level, (**D**) omega-3%, (**E**) DHA level, and (**F**) DHA% before and after treatment with JAKi or TCZ. Rx.: treatment. Data in Figure 2C–F are presented as box-plot diagrams, with the box encompassing the 25th percentile (lower bar) to the 75th percentile (upper bar). The horizontal line within the box indicates median value respectively for each group. * *p* < 0.05, ** *p* < 0.005, *** *p* < 0.001, was determined by the Wilcoxon signed rank test. JAKi: Janus kinase inhibitors; TCZ: tocilizumab; PUFAs: polyunsaturated fatty acids; DHA: docosahexaenoic acid; ns: no significance.

Using ^1^H-NMR technology, we analyzed the changes of serum levels of lipid metabolites in JAKi-treated and TCZ-treated patients at baseline and at week 24. As illustrated in Supplemental Table S2, a significant change of 13 markers of 18 analyzed fatty acids was observed in JAKi-treated patients, while not found in TCZ-treated patients. As shown in the volcano plots with the presentation of the fold change and p-value (Figure 2A,B), the most obvious changes were DHA, omega-3, omega-3%, and DHA% in JAKi-treated patients, but no significant change in TCZ-treated patients. Based on these findings, we focus on the four significant markers for the subsequent comparison. As shown in Figure 2C,D, omega–3 PUFAs levels and the ratio of omega–3 PUFAs to total fatty acids were significantly increased in JAKi-treated patients compared with baseline levels (median 0.55 mmol/L, IQR 0.47–0.67 mmol/L vs. 0.71 mmol/L, IQR 0.56–0.89 mmol/L, *p* = 0.0005; and 4.13%, IQR 3.75–5.03% vs. 4.92%, IQR 4.28–6.09%, *p* = 0.0056; respectively). JAKi-treated patients also had significantly increased levels of DHA and ratio of DHA to total fatty acids compared with baseline levels (median 0.29 mmol/L, IQR 0.27–0.34 mmol/L vs. 0.35 mmol/L, IQR 0.30–0.42 mmol/L, *p* = 0.0004; and 2.31%, IQR 2.02–2.59% vs. 2.51%, IQR 2.31–2.96%, *p* = 0.0139; respectively) (Figure 2E,F). However, the changes of omega-3 PUFAs or DHA levels, as well as the ratio of omega-3 PUFAs or DHA to total fatty acids, were all nonsignificant in TCZ-treated patients (Figure 2).

**Figure 3:**
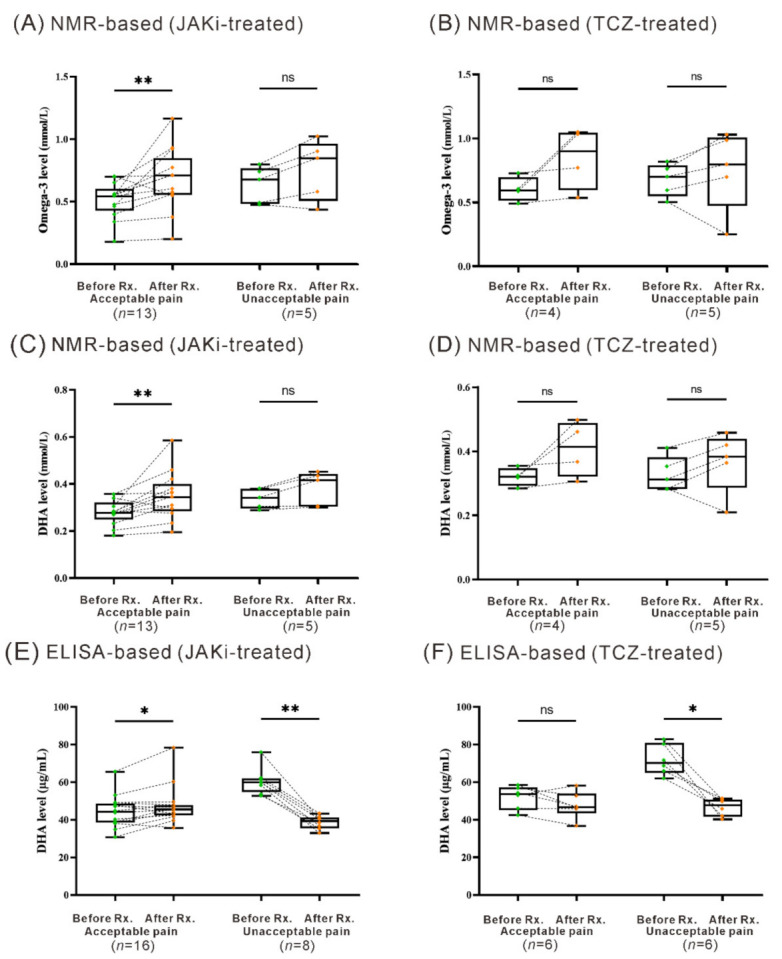
Change of serum levels of omega-3 PUFAs and DHA in JAKi-treated and TCZ-treated patients with acceptable pain or unacceptable pain assessed at week 24. The comparison of serum levels of omega-3 determined by NMR-based lipid/metabolomics in (**A**) JAKi-treated or (**B**) TCZ-treated patients with acceptable pain or unacceptable pain assessed at week 24. The comparison of serum levels of DHA determined by NMR-based lipid/metabolomics in (**C**) JAKi-treated or (**D**) TCZ-treated patients with acceptable pain or unacceptable pain assessed at week 24. The comparison of serum DHA levels determined by ELISA in (**E**) JAKi-treated and (**F**) TCZ-treated patients with acceptable pain or unacceptable pain assessed at week 24. Data are presented as box-plot diagrams, with the box encompassing the 25th percentile (lower bar) to the 75th percentile (upper bar). The horizontal line within the box indicates median value respectively for each group. * *p* < 0.05, ** *p* < 0.005, was determined by the Wilcoxon signed rank test. NMR-based: ^1^H-nuclear magnetic resonance-based lipid/metabolomics; JAKi: Janus kinase inhibitors; TCZ: tocilizumab; PUFAs: polyunsaturated fatty acids; DHA: docosahexaenoic acid; ns: no significance.

For JAKi-treated patients, we also revealed significantly increased levels of omega-3 PUFAs and DHA in those who reported “acceptable pain” after the treatment (median 0.54 mmol/L, IQR 0.46–0.56 mmol/L vs. 0.71 mmol/L, IQR 0.56–0.77 mmol/L, *p* = 0.0034; median 0.28 mmol/L, IQR 0.27–0.30 mmol/L vs. 0.35 mmol/L, IQR 0.30–0.38 mmol/L, *p* = 0.0061), but a nonsignificant change was observed in those reporting “unacceptable pain” after treatment (Figure 3A,C). However, there nonsignificant change observed in TCZ-treated patients (Figure 3B,D).

### 3.3. Change of Serum DHA Levels Determined by ELISA in Patients Treated with Six Months’ JAKi or TCZ

Based on the ^1^H NMR-based lipid/metabolomics results, we performed a replication study using another assay, ELISA, for determining a pure compound DHA. Our results showed a trend of positive correlation between baseline DHA levels from the NMR-based assay and those from ELISA-based assay (r = 0.327, *p* = 0.096) in RA patients. As shown in Figure 3E, significantly increased DHA levels (median 44.30 µg/mL, IQR 38.77–48.10 µg/mL vs. 45.61 µg/mL, IQR 42.52–47.61 µg/mL, *p* = 0.028) were observed in JAKi–treated patients who reported “acceptable pain” after the treatment, while decreased DHA levels in those with “unacceptable pain” despite a low disease activity (median 59.90 µg/mL, IQR 57.17–61.58 µg/mL vs. 39.39 µg/mL, IQR 36.39–40.73 µg/mL, *p* = 0.0078). In TCZ-treated patients, the change of DHA levels in “acceptable pain” patients was nonsignificant, in contrast to decreased DHA levels in “unacceptable pain” patients (Figure 3F, median 70.21 µg/mL, IQR 66.63–78.13 µg/mL vs. 47.75 µg/mL, IQR 42.97–50.45 µg/mL, *p* = 0.0313).

### 3.4. Correlation between the Change of DHA Levels and the Decrement of Pain Score or DAS28 Score in JAKi-Treated Patients

**Figure 4:**
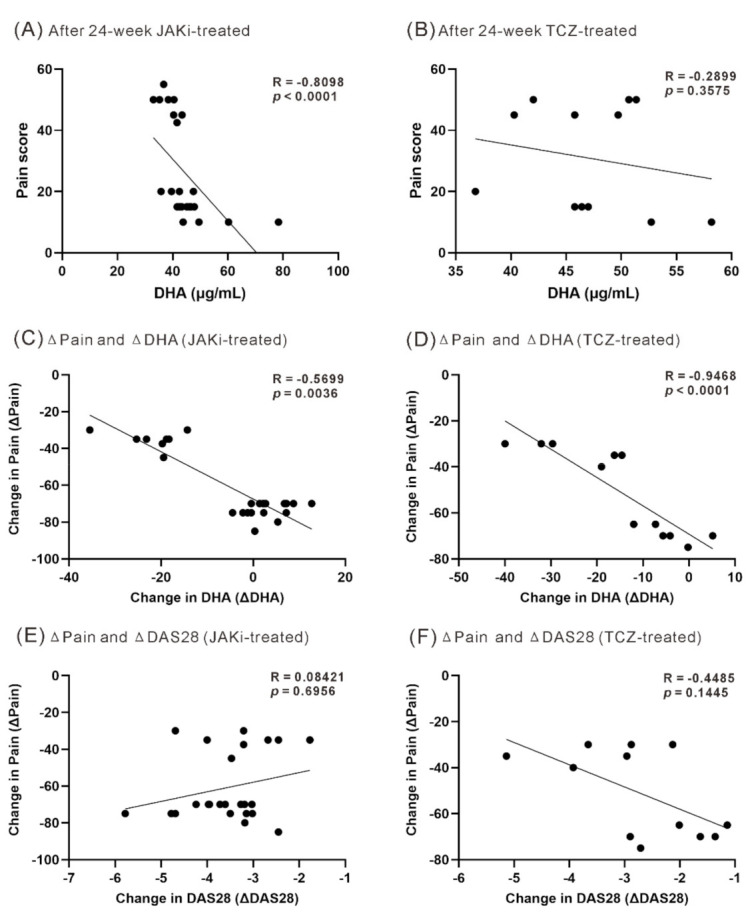
The correlation between DHA levels and pain scores after treatment or between pain decrement and the change of DHA levels or DAS28 scores. The correlation between serum DHA levels and pain scores assessed at week 24 in (**A**) JAKi-treated or (**B**) TCZ-treated patients. The correlation between the decrement of pain scores and the change of DHA levels in (**C**) JAKi-treated or (**D**) TCZ-treated patients. The correlation between the decrement of pain scores and the decrement of disease activity (DAS28 scores) in (**E**) JAKi-treated or (**F**) TCZ-treated patients. The correlation coefficient (R-value) was calculated using the nonparametric Spearman’s rank correlation test. JAK: Janus kinase inhibitors; TCZ: tocilizumab; DAS28: disease activity score for 28-joints.

There was an inverse correlation between serum DHA levels and patients’ reporting pain scores after six months’ treatment with JAKi (r = −0.810, *p* <0.001, Figure 4A), but a nonsignificant correlation in TCZ-treated patients (Figure 4B). In addition, a significant correlation was observed between the decrement of pain score and the change of DHA levels in JAKi-treated patients (r = −0.5699, *p* = 0.0036, Figure 4C) or TCZ-treated patients (r = −0.9468, *p* <0.0001, Figure 4D). However, there was no significant correlation between pain decrement and the change of disease activity (DAS28 scores) in JAKi-treated patients (Figure 4E) and TCZ-treated patients (Figure 4F). There was no significant correlation between the decrement of ESR and the change of omega-3 FA levels in JAKi-treated or TCZ-treated patients (r = 0.049, *p* = 0.848 or r = −0.367, *p* = 0.336, respectively), or between the decrement of CRP and the change of omega-3 FA levels in JAKi-treated or TCZ-treated patients (r = 0.115, *p* = 0.651 or r = 0.217, *p* = 0.581, respectively). There was also no significant correlation between the decrement of ESR and the change of DHA levels in JAKi-treated or TCZ-treated patients (r = −0.241, *p* = 0.256 or r = −0.245, *p* = 0.439, respectively), or between the decrement of CRP and the change of DHA levels in JAKi-treated or TCZ-treated patients (r = 0.018, *p* = 0.934 or r = 0.329, *p* = 0.297, respectively).

### 3.5. Linear Regression Analysis for Pain Decrement after JAKi Treatment

Using a linear regression analysis with pain decrement as the dependent variable, only the change in DHA levels and gender reached the set *p*-value (Table 2). The multivariate regression analysis revealed the change in DHA levels was significantly associated with the pain decrement.

## 4. Discussion

JAKi are known to have an additional pain reduction effect in RA patients, but the mechanism has not been fully elucidated. Similar to the results from a matching-adjusted indirect comparison study [22], the pain decrement at week 24 is greater in our JAKi-treated patients than in those treated with TCZ. Given that omega-3 PUFAs and DHA also showed a beneficial effect of pain reduction [26–30], we examined the changes of their serum levels in RA treated with JAKi or TCZ. Using the ^1^H NMR-based lipid/metabolomics, we revealed for the first time that serum levels of omega-3 PUFAs and DHA were significantly increased in patients treated with JAKi. The replicative ELISA results also showed increased DHA levels in JAKi-treated patients who reported acceptable pain (VAS pain ≤ 20), while decreased DHA levels in those reporting unacceptable pain. Besides, we revealed a significant correlation between the change of DHA levels and pain decrement in JAKi-treated patients. Regarding TCZ-treated patients, there was no significant change in DHA levels in those reporting acceptable pain, and the serum omega-3 PUFAs or DHA levels did not show significant changes even in patients with low disease activity or remission. Given that omega-3 PUFAs and DHA attenuate nociceptive pain through central and peripheral action [26,27,28], the additional pain reduction effect of JAKi may be linked to these lipid metabolites.

Although both JAKi and TCZ induced rapid normalization of inflammatory markers in our RA patients, the use of JAKi caused greater pain reduction than TCZ treatment, indicating that JAKi therapy enhanced pain relief beyond inflammation control. Many other studies, including matching-adjusted indirect comparison, also showed that JAKi could additionally provide pain relief, which was not seen in other biologics such as TCZ [20,21,22]. Like IL-6 signaling, which is integral to the pain mediated by sensory neurons, and interferon (IFN)-γ, which may potentiate neuropathic pain [36,37,38], multiple type I and II cytokines regulated by the JAK/STAT pathway have also been implicated in pain modulation in RA [9,10,39,40]. Thus, JAKi may provide better pain relief by inhibiting the JAK/STAT pathway and the related multiple cytokines, including IL-6 and IFN-γ. However, the mechanism underlying JAKi-related pain relief has yet been fully explored.

Regarding the anti-inflammatory and analgesic properties of omega-3 PUFAs and DHA [26,27,28], several previous studies, including a systemic review and meta-analysis, revealed that both lipid metabolites could significantly reduce pain in RA patients compared with baseline or placebo [29,30]. Therefore, omega-3 PUFAs or DHA dietary supplements might reduce pain and inflammation in RA [29,30,41,42]. In the first half of our study where we aimed to explore the association between pain reduction and the changes in lipid metabolites, the ^1^H NMR-based lipid/metabolomics results showed significantly increased omega-3 PUFAs and DHA levels in patients treated with JAKi, which was not found in TCZ-treated patients. Similarly, the omega-3 PUFAs and DHA levels were significantly increased in patients with acceptable pain after 24-week JAKi treatment, which was also not observed in TCZ-treated patients. Furthermore, we replicated NMR-based lipidomics results of DHA by using ELISA, and similarly revealed increased DHA levels in JAKi-treated patients who reported acceptable pain, in contrast to decreased DHA levels in those with unacceptable pain. Our results support the finding of previous studies that the dietary supplements of omega-3 PUFAs or DHA may reduce pain and inflammation in RA [29,30,41,42]. The different effects of JAKi and TCZ on omega-3 PUFAs and DHA may be related to their disparate mechanisms of action, different pharmacokinetics, and pharmacodynamics.

In the present study, we revealed an inverse correlation between serum DHA levels and patients’ reporting pain scores after six months’ JAKi treatment with inflammation control. Furthermore, an inverse correlation between the pain decrement and DHA levels increment was observed in JAKi-treated patients. Using a multivariate regression analysis, only the change of DHA levels could predict the pain reduction in patients after six months’ JAKi treatment. It suggests that JAKi-related pain relief may be at least partly linked to the increased DHA levels. Interestingly, there was no significant correlation between the pain decrement and the change of disease activity (DAS28 scores) in our JAKi-treated patients, showing a discrepancy between the degree of RA inflammation and the severity of perceived pain. Given that omega-3 PUFAs and DHA can attenuate nociceptive pain through central and peripheral actions [26,27,28], the pain reduction effect associated with JAKi therapy may be partly related to elevated lipid metabolites levels rather than simply inflammatory control. These observations resonate with a review article focusing on noninflammatory pain control in rheumatic patients [43].

Despite the novel findings in the present study, there were still some limitations. First, this study is a pilot study enrolling a small number of biologic-naïve patients, which may reduce the statistical power. Out of ethical concerns, the enrolled RA patients were allowed to use corticosteroids or methotrexate, which may affect lipid metabolism. Nevertheless, we revealed that JAKi therapy might promote pain relief through increasing omega-3 PUFAs and DHA levels, although a large prospective study should be conducted to confirm our findings.

## 5. Conclusions

We used NMR-based lipidomics and ELISA assays to provide biochemical evidence for the possible mechanism underlying the pain reduction effect of JAKi in RA patients: the increased omega-3 fatty acids and DHA levels. Dietary supplements of omega-3 PUFAs and DHA may have beneficial effect on pain reduction despite inflammation control in RA.

## Figures and Tables

**Table 1:** Demographic, clinical characteristics, and laboratory data in rheumatoid arthritis (RA) patients treated with Janus kinase inhibitors (JAKi) or tocilizumab (TCZ) **^a^**.

	JAKi-Treated Patients (*n* = 24)	TCZ-Treated Patients (*n* = 12)
Age at entry, years	60.5 (55.5–65.3)	59.0 (50.3–64.0)
Age at disease onset, years	53.0 (48.0–59.3)	51.0 (44.0–56.5)
Disease duration, years	5.0 (4.0–6.5)	7.0 (4.8–8.5)
Proportion of women	18 (75.0%)	10 (83.3%)
BMI, kg/m^2^	23.6 (21.1–25.8)	22.7 (21.1–25.7)
RF positivity, at baseline	15 (62.5%)	8 (66.7%)
ACPA positivity, at baseline	15 (62.5%)	9 (75.0%)
DAS28 at baseline	6.77 (6.23–7.13)	7.04 (5.98–7.36)
DAS28 at week 24	3.12 (3.05–3.40) **	3.14 (3.08–3.30) **
Change of DAS28 (ΔDAS28)	3.37 (3.12–3.98)	3.64 (2.94–4.10)
Tender joint count at baseline	15 (10–22)	15 (12–19)
Tender joint count at week 24	2 (2–3) **	4 (3–9) **
Swollen joint count at baseline	10 (5–12)	9 (7–14)
Swollen joint count at week 24	2 (1–3) **	2 (2–5) **
Pain scores at baseline ^b^	87.5 (85.0–90.9)	80.0 (80.0–85.0)
Pain scores at week 24 ^b^	17.5 (15.0–45.0) **	32.5 (15.0–46.3) **
Change of pain scores (Δpain scores)	70.0 (36.9–75.0)	52.5 (33.8–70.0)
ESR, mm/1st h, at baseline	32 (23–49)	37 (23–46)
ESR, mm/1st h, at week 24	16 (11–24) **	8 (4–10) *
Change of ESR (ΔESR), mm/1st h	21 (7–30)	30 (18–38)
CRP, mg/dL, at baseline	1.79 (0.98–2.75)	1.61 (0.78–3.93)
CRP, mg/dL, at week 24	0.17 (0.06–0.57) **	0.02 (0.02–0.08) *
Change of CRP (ΔCRP), mg/dL	1.14 (0.46–2.53)	1.59 (0.76–3.65)
WBC (×10^3^/mm^3^) at baseline	7.6 (6.5–9.9)	5.8 (4.9–7.8)
WBC (×10^3^/mm^3^) at week 24	6.4 (5.4–8.4) *	5.0 (4.6–5.8)
Neutrophil (%) at baseline	72.6 (60.3–74.9)	60.2 (51.5–70.4)
Neutrophil (%) at week 24	62.8 (58.8–65.9)	47.5 (41.3–53.1) *
Lymphocyte (%) at baseline	19.0 (15.5–21.5)	26.7 (19.2–31.5)
Lymphocyte (%) at week 24	26.7 (24.3–28.6)	34.3 (32.7–38.1)
TC, mg/dL, at baseline	179.5 (161.8–208.8)	206.0 (181.5–221.5)
TC, mg/dL, at week 24	193.0 (175.0–213.5)	212.0 (183.3–230.3)
HDL-C, mg/dL, at baseline	56.5 (52.2–67.0)	58.7 (46.6–70.7)
HDL-C, mg/dL, at week 24	60.4 (51.6–71.0)	56.1 (48.5–62.9)
TG, mg/dL, at baseline	79.0 (51.8–127.5)	81.5 (69.8–123.3)
TG, mg/dL, at week 24	83.0 (59.5–131.0)	113.0 (82.0–148.0)
LDL-C, mg/dL, at baseline	104.5 (88.9–123.6)	123.7 (103.1–137.3)
LDL-C, mg/dL, at week 24	103.6 (97.6–113.3)	131.6 (106.2–142.2)
Concomitant corticosteroids	10 (41.7%)	6 (50.0%)
Concomitant methotrexate	15 (62.5%)	8 (66.7%)
Hypertension	6 (25.0%)	4 (33.3%)
Diabetes mellitus	2 (8.3%)	0 (0.0%)
Ever smoking	3 (12.5%)	1 (8.3%)

**^a^** Data are presented as median (interquartile range, IQR) or number (%). **^b^** Pain scores based on patient’s assessment using the 100 mm visual analogue scales. BMI: body mass index; RF: rheumatoid factor; ACPA: anti-citrullinated peptide antibodies; DAS28: disease activity score for 28-joints; ESR: erythrocyte sedimentation rate; CRP: C-reactive protein; WBC: white blood cells; TC: total cholesterol; HDL-C: high-density lipoprotein cholesterol; TG: triglyceride; LDL-C: low-density lipoprotein cholesterol. * *p* < 0.01, ** *p* < 0.001, vs. before treatment (at baseline), as determined by Wilcoxon signed rank test.

**Table 2:** Linear regression analysis for pain decrement after JAKi treatment.

**Univariate Regression Analysis**				
	**B**	**95% CI**		***p* Value**
Change in DHA	−1.284	−1.581	−0.988	<0.0001
Change in ESR	0.383	−0.066	0.831	0.0904
Change in CRP	0.867	−1.366	3.099	0.4285
Change in DAS28	5.215	−3.994	14.425	0.2528
Age at entry	−0.319	−1.066	0.429	0.3862
Gender	20.139	3.506	36.772	0.0199
**Multiple Regression Analysis**				
	**B**	**95% CI**		***p* value**
Change in DHA	−1.148	−1.532	−0.765	<0.0001
Change in ESR	0.057	−0.295	0.409	0.7362
Change in CRP	0.399	−0.928	1.726	0.5327
Change in DAS28	−0.413	−7.358	6.532	0.9013
Age at entry	−0.101	−0.505	0.303	0.6046
Gender	8.250	−2.538	19.038	0.1245

## Data Availability

The datasets used and/or analyzed during the current study are available from the corresponding author on reasonable request.

## Supplementary Materials

The following are available online at https://www.mdpi.com/article/10.3390/nu13093050/s1, Table S1: The whole li-pid/metabolomics based on 1H-NMR analysis; Table S2: The fatty acids-related markers deter-mined by 1H-NMR-based lipid/metabolomics and their fold change in rheumatoid arthritis pa-tients treated with Janus kinase inhibitors (JAKi) or tocilizumab (TCZ).
