# Supplementary material for: Increased Levels of Omega-3 Fatty Acids and DHA Are Linked to Pain Reduction in Rheumatoid Arthritis Patients Treated with Janus Kinase Inhibitors
Source: Nutrients. 2021 Aug 30;13(9):3050. doi: 10.3390/nu13093050 (PMC8465317; doi:10.3390/nu13093050)
Supplement: Supplementary file 1 [file nutrients-13-03050-s001.zip › nutrients-1333193-supplementary.pdf]

Table S1. The fatty acids-related markers determined by ^{1}H-NMR-based lipid/metabolomics and their fold change in rheumatoid arthritis patients treated with Janus kinase inhibitors (JAKi) or tocilizumab (TCZ).

	**JAKi-treated patients (n=18)**			**TCZ-treated patients (n=9)**		
	Fold change	p value	p value (FDR)	Fold change	p value	p value (FDR)
DHA	1.0765	0.0074	0.0334	1.0905	0.3594	0.9010
Omega-3	1.0255	0.0009	0.0334	1.0217	0.0957	0.8562
PUFA	1.2774	0.0010	0.0334	1.2651	0.0547	0.8562
Unsaturated FA	1.0810	0.0077	0.0334	1.0850	0.3008	0.8643
Omega-3 % [a]	1.0995	0.0013	0.0334	1.1037	0.1641	0.8562
Total-FA	1.0495	0.0737	0.1269	1.0785	0.2500	0.8562
LA	1.0642	0.0186	0.0481	1.0786	0.4258	0.9010
Omega-6	1.0938	0.0092	0.0349	1.1027	0.2600	0.8643
SFA	1.2097	0.0013	0.0334	1.1913	0.0742	0.8562
DHA % [a]	1.1856	0.0053	0.0334	1.1401	0.1289	0.8562
PUFA/MUFA	1.0042	0.8446	0.8762	0.9981	0.9055	0.9936
PUFA % [a]	1.0205	0.0345	0.0725	1.0128	0.2131	0.8562
MUFA % [a]	0.9745	0.0235	0.0533	0.9900	0.4390	0.9010
Omega-6/Omega-3	0.9885	0.5071	0.5924	0.9873	0.4065	0.9010
MUFA	1.0147	0.6164	0.6879	1.0148	0.6241	0.9010
LA % [a]	1.1228	0.0553	0.1056	1.0877	0.2357	0.8562
SFA % [a]	1.0475	0.0158	0.0428	1.0281	0.4768	0.9010
Omega-6 % [a]	0.9051	0.0599	0.1102	0.9533	0.1921	0.8562

Total-FA: Total fatty acids; PUFA: Polyunsaturated fatty acids; MUFA: monounsaturated fatty acids; SFA: Saturated fatty acids; LA: Linoleic acid; DHA: Docosahexaenoic acid.

[a] The % means the ration with total fatty acids.

Table S2. The whole lipid/metabolomics based on ^{1}H-NMR analysis.

Marker	Unit	Marker description
Total-C	mmol/L	Total cholesterol
non-HDL-C	mmol/L	Total cholesterol minus HDL-C
Remnant-C	mmol/L	Remnant cholesterol (non-HDL, non-LDL -cholesterol)
VLDL-C	mmol/L	VLDL cholesterol
Clinical LDL-C	mmol/L	Clinical LDL cholesterol
LDL-C	mmol/L	LDL cholesterol
HDL-C	mmol/L	HDL cholesterol
Total-TG	mmol/L	Total triglycerides
VLDL-TG	mmol/L	Triglycerides in VLDL
LDL-TG	mmol/L	Triglycerides in LDL
HDL-TG	mmol/L	Triglycerides in HDL
Total-PL	mmol/L	Total phospholipids in lipoprotein particles
VLDL-PL	mmol/L	Phospholipids in VLDL
LDL-PL	mmol/L	Phospholipids in LDL
HDL-PL	mmol/L	Phospholipids in HDL
Total-CE	mmol/L	Total esterified cholesterol
VLDL-CE	mmol/L	Cholesteryl esters in VLDL
LDL-CE	mmol/L	Cholesteryl esters in LDL
HDL-CE	mmol/L	Cholesteryl esters in HDL
Total-FC	mmol/L	Total free cholesterol
VLDL-FC	mmol/L	Free cholesterol in VLDL
LDL-FC	mmol/L	Free cholesterol in LDL
HDL-FC	mmol/L	Free cholesterol in HDL
Total-L	mmol/L	Total lipids in lipoprotein particles
VLDL-L	mmol/L	Total lipids in VLDL
LDL-L	mmol/L	Total lipids in LDL
HDL-L	mmol/L	Total lipids in HDL
Total-P	mmol/L	Total concentration of lipoprotein particles
VLDL-P	mmol/L	Concentration of VLDL particles
LDL-P	mmol/L	Concentration of LDL particles
HDL-P	mmol/L	Concentration of HDL particles
VLDL size	nm	Average diameter for VLDL particles
LDL size	nm	Average diameter for LDL particles
HDL size	nm	Average diameter for HDL particles
Phosphoglyc	mmol/L	Phosphoglycerides

TG/PG	ratio	Ratio of triglycerides to phosphoglycerides
Cholines	mmol/L	Total cholines
Phosphatidylc	mmol/L	Phosphatidylcholines
Sphingomyelins	mmol/L	Sphingomyelins
ApoB	g/L	Apolipoprotein B
ApoA1	g/L	Apolipoprotein A1
ApoB/ApoA1	ratio	Ratio of apolipoprotein B to apolipoprotein A1
Total-FA	mmol/L	Total fatty acids
Unsaturation	degree	Degree of unsaturation
Omega-3	mmol/L	Omega-3 fatty acids
Omega-6	mmol/L	Omega-6 fatty acids
PUFA	mmol/L	Polyunsaturated fatty acids
MUFA	mmol/L	Monounsaturated fatty acids
SFA	mmol/L	Saturated fatty acids
LA	mmol/L	Linoleic acid
DHA	mmol/L	Docosahexaenoic acid
Omega-3 %	%	Ratio of omega-3 fatty acids to total fatty acids
Omega-6 %	%	Ratio of omega-6 fatty acids to total fatty acids
PUFA %	%	Ratio of polyunsaturated fatty acids to total fatty acids
MUFA %	%	Ratio of monounsaturated fatty acids to total fatty acids
SFA %	%	Ratio of saturated fatty acids to total fatty acids
LA %	%	Ratio of linoleic acid to total fatty acids
DHA %	%	Ratio of docosahexaenoic acid to total fatty acids
PUFA/MUFA	ratio	Ratio of polyunsaturated fatty acids to monounsaturated fatty acids
Omega-6/Omega-3	ratio	Ratio of omega-6 fatty acids to omega-3 fatty acids
Ala	mmol/L	Alanine
Gln	mmol/L	Glutamine
Gly	mmol/L	Glycine
His	mmol/L	Histidine
Total BCAA	mmol/L	Total concentration of branched-chain amino acids (leucine + isoleucine + valine)
Ile	mmol/L	Isoleucine
Leu	mmol/L	Leucine
Val	mmol/L	Valine
Phe	mmol/L	Phenylalanine

Tyr	mmol/L	Tyrosine
Glucose	mmol/L	Glucose
Lactate	mmol/L	Lactate
Pyruvate	mmol/L	Pyruvate
Citrate	mmol/L	Citrate
Glycerol	mmol/L	Glycerol
bOHbutyrate	mmol/L	3-Hydroxybutyrate
Acetate	mmol/L	Acetate
Acetoacetate	mmol/L	Acetoacetate
Acetone	mmol/L	Acetone
Creatinine	μmol/L	Creatinine
Albumin	g/L	Albumin
GlycA	mmol/L	Glycoprotein acetyls
XXL-VLDL-P	mmol/L	Concentration of chylomicrons and extremely large VLDL particles
XXL-VLDL-L	mmol/L	Total lipids in chylomicrons and extremely large VLDL
XXL-VLDL-PL	mmol/L	Phospholipids in chylomicrons and extremely large VLDL
XXL-VLDL-C	mmol/L	Cholesterol in chylomicrons and extremely large VLDL
XXL-VLDL-CE	mmol/L	Cholesteryl esters in chylomicrons and extremely large VLDL
XXL-VLDL-FC	mmol/L	Free cholesterol in chylomicrons and extremely large VLDL
XXL-VLDL-TG	mmol/L	Triglycerides in chylomicrons and extremely large VLDL
XL-VLDL-P	mmol/L	Concentration of very large VLDL particles
XL-VLDL-L	mmol/L	Total lipids in very large VLDL
XL-VLDL-PL	mmol/L	Phospholipids in very large VLDL
XL-VLDL-C	mmol/L	Cholesterol in very large VLDL
XL-VLDL-CE	mmol/L	Cholesteryl esters in very large VLDL
XL-VLDL-FC	mmol/L	Free cholesterol in very large VLDL
XL-VLDL-TG	mmol/L	Triglycerides in very large VLDL
L-VLDL-P	mmol/L	Concentration of large VLDL particles
L-VLDL-L	mmol/L	Total lipids in large VLDL
L-VLDL-PL	mmol/L	Phospholipids in large VLDL
L-VLDL-C	mmol/L	Cholesterol in large VLDL
L-VLDL-CE	mmol/L	Cholesteryl esters in large VLDL
L-VLDL-FC	mmol/L	Free cholesterol in large VLDL

L-VLDL-TG	mmol/L	Triglycerides in large VLDL
M-VLDL-P	mmol/L	Concentration of medium VLDL particles
M-VLDL-L	mmol/L	Total lipids in medium VLDL
M-VLDL-PL	mmol/L	Phospholipids in medium VLDL
M-VLDL-C	mmol/L	Cholesterol in medium VLDL
M-VLDL-CE	mmol/L	Cholesteryl esters in medium VLDL
M-VLDL-FC	mmol/L	Free cholesterol in medium VLDL
M-VLDL-TG	mmol/L	Triglycerides in medium VLDL
S-VLDL-P	mmol/L	Concentration of small VLDL particles
S-VLDL-L	mmol/L	Total lipids in small VLDL
S-VLDL-PL	mmol/L	Phospholipids in small VLDL
S-VLDL-C	mmol/L	Cholesterol in small VLDL
S-VLDL-CE	mmol/L	Cholesteryl esters in small VLDL
S-VLDL-FC	mmol/L	Free cholesterol in small VLDL
S-VLDL-TG	mmol/L	Triglycerides in small VLDL
XS-VLDL-P	mmol/L	Concentration of very small VLDL particles
XS-VLDL-L	mmol/L	Total lipids in very small VLDL
XS-VLDL-PL	mmol/L	Phospholipids in very small VLDL
XS-VLDL-C	mmol/L	Cholesterol in very small VLDL
XS-VLDL-CE	mmol/L	Cholesteryl esters in very small VLDL
XS-VLDL-FC	mmol/L	Free cholesterol in very small VLDL
XS-VLDL-TG	mmol/L	Triglycerides in very small VLDL
IDL-P	mmol/L	Concentration of IDL particles
IDL-L	mmol/L	Total lipids in IDL
IDL-PL	mmol/L	Phospholipids in IDL
IDL-C	mmol/L	Cholesterol in IDL
IDL-CE	mmol/L	Cholesteryl esters in IDL
IDL-FC	mmol/L	Free cholesterol in IDL
IDL-TG	mmol/L	Triglycerides in IDL
L-LDL-P	mmol/L	Concentration of large LDL particles
L-LDL-L	mmol/L	Total lipids in large LDL
L-LDL-PL	mmol/L	Phospholipids in large LDL
L-LDL-C	mmol/L	Cholesterol in large LDL
L-LDL-CE	mmol/L	Cholesteryl esters in large LDL
L-LDL-FC	mmol/L	Free cholesterol in large LDL
L-LDL-TG	mmol/L	Triglycerides in large LDL
M-LDL-P	mmol/L	Concentration of medium LDL particles
M-LDL-L	mmol/L	Total lipids in medium LDL

M-LDL-PL	mmol/L	Phospholipids in medium LDL
M-LDL-C	mmol/L	Cholesterol in medium LDL
M-LDL-CE	mmol/L	Cholesteryl esters in medium LDL
M-LDL-FC	mmol/L	Free cholesterol in medium LDL
M-LDL-TG	mmol/L	Triglycerides in medium LDL
S-LDL-P	mmol/L	Concentration of small LDL particles
S-LDL-L	mmol/L	Total lipids in small LDL
S-LDL-PL	mmol/L	Phospholipids in small LDL
S-LDL-C	mmol/L	Cholesterol in small LDL
S-LDL-CE	mmol/L	Cholesteryl esters in small LDL
S-LDL-FC	mmol/L	Free cholesterol in small LDL
S-LDL-TG	mmol/L	Triglycerides in small LDL
XL-HDL-P	mmol/L	Concentration of very large HDL particles
XL-HDL-L	mmol/L	Total lipids in very large HDL
XL-HDL-PL	mmol/L	Phospholipids in very large HDL
XL-HDL-C	mmol/L	Cholesterol in very large HDL
XL-HDL-CE	mmol/L	Cholesteryl esters in very large HDL
XL-HDL-FC	mmol/L	Free cholesterol in very large HDL
XL-HDL-TG	mmol/L	Triglycerides in very large HDL
L-HDL-P	mmol/L	Concentration of large HDL particles
L-HDL-L	mmol/L	Total lipids in large HDL
L-HDL-PL	mmol/L	Phospholipids in large HDL
L-HDL-C	mmol/L	Cholesterol in large HDL
L-HDL-CE	mmol/L	Cholesteryl esters in large HDL
L-HDL-FC	mmol/L	Free cholesterol in large HDL
L-HDL-TG	mmol/L	Triglycerides in large HDL
M-HDL-P	mmol/L	Concentration of medium HDL particles
M-HDL-L	mmol/L	Total lipids in medium HDL
M-HDL-PL	mmol/L	Phospholipids in medium HDL
M-HDL-C	mmol/L	Cholesterol in medium HDL
M-HDL-CE	mmol/L	Cholesteryl esters in medium HDL
M-HDL-FC	mmol/L	Free cholesterol in medium HDL
M-HDL-TG	mmol/L	Triglycerides in medium HDL
S-HDL-P	mmol/L	Concentration of small HDL particles
S-HDL-L	mmol/L	Total lipids in small HDL
S-HDL-PL	mmol/L	Phospholipids in small HDL
S-HDL-C	mmol/L	Cholesterol in small HDL
S-HDL-CE	mmol/L	Cholesteryl esters in small HDL

S-HDL-FC	mmol/L	Free cholesterol in small HDL
S-HDL-TG	mmol/L	Triglycerides in small HDL
XXL-VLDL-PL %	%	Phospholipids to total lipids ratio in chylomicrons and extremely large VLDL
XXL-VLDL-C %	%	Cholesterol to total lipids ratio in chylomicrons and extremely large VLDL
XXL-VLDL-CE %	%	Cholesteryl esters to total lipids ratio in chylomicrons and extremely large VLDL
XXL-VLDL-FC %	%	Free cholesterol to total lipids ratio in chylomicrons and extremely large VLDL
XXL-VLDL-TG %	%	Triglycerides to total lipids ratio in chylomicrons and extremely large VLDL
XL-VLDL-PL %	%	Phospholipids to total lipids ratio in very large VLDL
XL-VLDL-C %	%	Cholesterol to total lipids ratio in very large VLDL
XL-VLDL-CE %	%	Cholesteryl esters to total lipids ratio in very large VLDL
XL-VLDL-FC %	%	Free cholesterol to total lipids ratio in very large VLDL
XL-VLDL-TG %	%	Triglycerides to total lipids ratio in very large VLDL
L-VLDL-PL %	%	Phospholipids to total lipids ratio in large VLDL
L-VLDL-C %	%	Cholesterol to total lipids ratio in large VLDL
L-VLDL-CE %	%	Cholesteryl esters to total lipids ratio in large VLDL
L-VLDL-FC %	%	Free cholesterol to total lipids ratio in large VLDL
L-VLDL-TG %	%	Triglycerides to total lipids ratio in large VLDL
M-VLDL-PL %	%	Phospholipids to total lipids ratio in medium VLDL
M-VLDL-C %	%	Cholesterol to total lipids ratio in medium VLDL
M-VLDL-CE %	%	Cholesteryl esters to total lipids ratio in medium VLDL
M-VLDL-FC %	%	Free cholesterol to total lipids ratio in medium VLDL

M-VLDL-TG %	%	Triglycerides to total lipids ratio in medium VLDL
S-VLDL-PL %	%	Phospholipids to total lipids ratio in small VLDL
S-VLDL-C %	%	Cholesterol to total lipids ratio in small VLDL
S-VLDL-CE %	%	Cholesteryl esters to total lipids ratio in small VLDL
S-VLDL-FC %	%	Free cholesterol to total lipids ratio in small VLDL
S-VLDL-TG %	%	Triglycerides to total lipids ratio in small VLDL
XS-VLDL-PL %	%	Phospholipids to total lipids ratio in very small VLDL
XS-VLDL-C %	%	Cholesterol to total lipids ratio in very small VLDL
XS-VLDL-CE %	%	Cholesteryl esters to total lipids ratio in very small VLDL
XS-VLDL-FC %	%	Free cholesterol to total lipids ratio in very small VLDL
XS-VLDL-TG %	%	Triglycerides to total lipids ratio in very small VLDL
IDL-PL %	%	Phospholipids to total lipids ratio in IDL
IDL-C %	%	Cholesterol to total lipids ratio in IDL
IDL-CE %	%	Cholesteryl esters to total lipids ratio in IDL
IDL-FC %	%	Free cholesterol to total lipids ratio in IDL
IDL-TG %	%	Triglycerides to total lipids ratio in IDL
L-LDL-PL %	%	Phospholipids to total lipids ratio in large LDL
L-LDL-C %	%	Cholesterol to total lipids ratio in large LDL
L-LDL-CE %	%	Cholesteryl esters to total lipids ratio in large LDL
L-LDL-FC %	%	Free cholesterol to total lipids ratio in large LDL
L-LDL-TG %	%	Triglycerides to total lipids ratio in large LDL
M-LDL-PL %	%	Phospholipids to total lipids ratio in medium LDL
M-LDL-C %	%	Cholesterol to total lipids ratio in medium LDL
M-LDL-CE %	%	Cholesteryl esters to total lipids ratio in medium LDL
M-LDL-FC %	%	Free cholesterol to total lipids ratio in medium LDL
M-LDL-TG %	%	Triglycerides to total lipids ratio in medium LDL
S-LDL-PL %	%	Phospholipids to total lipids ratio in small LDL
S-LDL-C %	%	Cholesterol to total lipids ratio in small LDL

S-LDL-CE %	%	Cholesteryl esters to total lipids ratio in small LDL
S-LDL-FC %	%	Free cholesterol to total lipids ratio in small LDL
S-LDL-TG %	%	Triglycerides to total lipids ratio in small LDL
XL-HDL-PL %	%	Phospholipids to total lipids ratio in very large HDL
XL-HDL-C %	%	Cholesterol to total lipids ratio in very large HDL
XL-HDL-CE %	%	Cholesteryl esters to total lipids ratio in very large HDL
XL-HDL-FC %	%	Free cholesterol to total lipids ratio in very large HDL
XL-HDL-TG %	%	Triglycerides to total lipids ratio in very large HDL
L-HDL-PL %	%	Phospholipids to total lipids ratio in large HDL
L-HDL-C %	%	Cholesterol to total lipids ratio in large HDL
L-HDL-CE %	%	Cholesteryl esters to total lipids ratio in large HDL
L-HDL-FC %	%	Free cholesterol to total lipids ratio in large HDL
L-HDL-TG %	%	Triglycerides to total lipids ratio in large HDL
M-HDL-PL %	%	Phospholipids to total lipids ratio in medium HDL
M-HDL-C %	%	Cholesterol to total lipids ratio in medium HDL
M-HDL-CE %	%	Cholesteryl esters to total lipids ratio in medium HDL
M-HDL-FC %	%	Free cholesterol to total lipids ratio in medium HDL
M-HDL-TG %	%	Triglycerides to total lipids ratio in medium HDL
S-HDL-PL %	%	Phospholipids to total lipids ratio in small HDL
S-HDL-C %	%	Cholesterol to total lipids ratio in small HDL
S-HDL-CE %	%	Cholesteryl esters to total lipids ratio in small HDL
S-HDL-FC %	%	Free cholesterol to total lipids ratio in small HDL
S-HDL-TG %	%	Triglycerides to total lipids ratio in small HDL
